# T2* Mapping of Placental Oxygenation to Estimate Fetal Cortical and Subcortical Maturation

**DOI:** 10.1001/jamanetworkopen.2024.0456

**Published:** 2024-02-27

**Authors:** Emily S. Nichols, Sarah Al-Saoud, Barbra de Vrijer, Charles A. McKenzie, Roy Eagleson, Sandrine de Ribaupierre, Emma G. Duerden

**Affiliations:** 1 Applied Psychology, Faculty of Education, Western University, London, Ontario, Canada; 2 Western Institute for Neuroscience, Western University, London, Ontario, Canada; 3 Obstetrics & Gynaecology, Schulich School of Medicine & Dentistry, Western University, London, Ontario, Canada; 4 Division of Maternal, Fetal and Newborn Health, Children’s Health Research Institute; 5 Medical Biophysics, Schulich School of Medicine & Dentistry, Western University, London, Ontario, Canada; 6 Electrical and Computer Engineering, Faculty of Engineering, Western University, London, Ontario, Canada; 7 Clinical Neurological Sciences, Schulich School of Medicine & Dentistry, Western University, London, Ontario, Canada; 8 Anatomy and Cell Biology, Schulich School of Medicine & Dentistry, Western University, London, Ontario, Canada; 9 Biomedical Engineering, Western University, London, Ontario, Canada

## Abstract

This cohort study investigates the association between T2* mapping of placental oxygenation and cortical and subcortical fetal brain volumes in typically developing fetuses scanned longitudinally in the third trimester.

## Introduction

Placental dysfunction is associated with a decrease in nutrients and oxygen to the fetus; the gestational age at which this happens varies depending on severity but is an important factor in outcome as it relates to when and which brain structures are most at risk. Evidence from Doppler ultrasonography of fetuses affected by severe placental dysfunction leading to intrauterine growth restriction (IUGR) suggests blood flow distribution occurs in a hierarchical manner. In IUGR, oxygenated blood is directed toward the brain, away from other fetal organs (except the fetal heart), a process referred to as brain sparing. Further evidence suggests that subcortical regions critical for homeostasis receive more blood flow, at the cost of cortical regions involved in higher-order functions. Although Doppler findings suggest that cortical regions show more variability to placental oxygenation changes, a Cochrane review found that the evidence was of moderate to low quality,^1^ indicating the need for more sensitive techniques to study how placental function affects the brain. Recent work has demonstrated an association between a magnetic resonance imaging (MRI)–based measure of placental oxygenation, transverse relaxation time (T2*), and birth weight,^2^ suggesting that T2* may similarly estimate variations in fetal brain development.

To determine whether placental MRI-based methods could provide a biomarker of fetal brain development, we investigated the association between placental T2* and cortical and subcortical fetal brain volumes in typically developing fetuses scanned longitudinally in the third trimester. We hypothesized that in fetuses with reduced placental oxygenation, cortical brain regions would show reduced volumes relative to subcortical regions.

## Methods

This prospective cohort study followed the STROBE reporting guideline^3^ and was approved by the research ethics board at Western University. Female participants were recruited from the community (December 2020 to August 2023) and provided written informed consent. Participants were imaged with MRI at 2 time points in the third trimester more than 2 weeks apart. 2D multiecho stack placental images and single-shot fast spin echo anatomical brain images were acquired using a 3T GE Discovery MRI scanner and 32-channel torso coil at Western University (see eMethods in Supplement 1 for full scanning parameters). Placental masks were manually drawn using FSLeyes version 1.6.1 (FMRIB Centre), and T2* values were calculated by fitting the mean signal within each mask as a function of echo time. The T2* values were standardized to adjust for gestational age.^4^ Cortical and subcortical volumes were extracted using previously published methods^5,6^ and converted to *z* scores (eMethods in Supplement 1). Volumes were submitted to linear mixed-effects (LME) analysis with an interaction between T2* values and region (cortical or subcortical), controlling for fetal sex, maternal age, socioeconomic status, and time point. Participant was included as a random effect. A significance threshold of *P* < .05 was used. Statistical analysis was performed using R statistical software version 4.0.3 (R Project for Statistical Computing) in September 2023.

## Results

Forty-nine female participants were scanned and second time point data were available for 38 participants due to birthing restrictions or inability to access the scanner (total = 87 scans) (Table). Placental T2* values decreased with gestational age (Figure A and B), and volumes increased with gestational age (Figure C). LME showed a significant interaction between T2* and region (*F*_1,117_ = 5.46; *P* = .02) (Figure D). Post hoc estimated marginal means of the trends indicated that the slope was larger in cortical vs subcortical regions (β = 0.09; *t*_114_ = 2.25; *P* = .03). There was a main effect of time point, but no other significant associations.

**Table.** Participant Characteristics

Measure	Mean (SD) [range]		
	Total	Time point 1	Time point 2
Participants, No.	49	49	38
Gestational age, y	32.81 (3.29) [26.9-39.3]	31.8 (3.28) [26.9-39.3]	34.1 (2.85) [28.9-39.1]
Fetal sex, No. (%)			
Female	21 (42.8)	NA	NA
Male	26 (53.1)	NA	NA
Unknown	2 (4)	NA	NA
Maternal age, y	31.98 (4.48)	NA	NA
Socioeconomic status^a^	29.80 (11.32)	NA	NA

Abbreviation: NA, not available.

^a^ Barratt Simplified Measure of Social Status.

**Figure.**
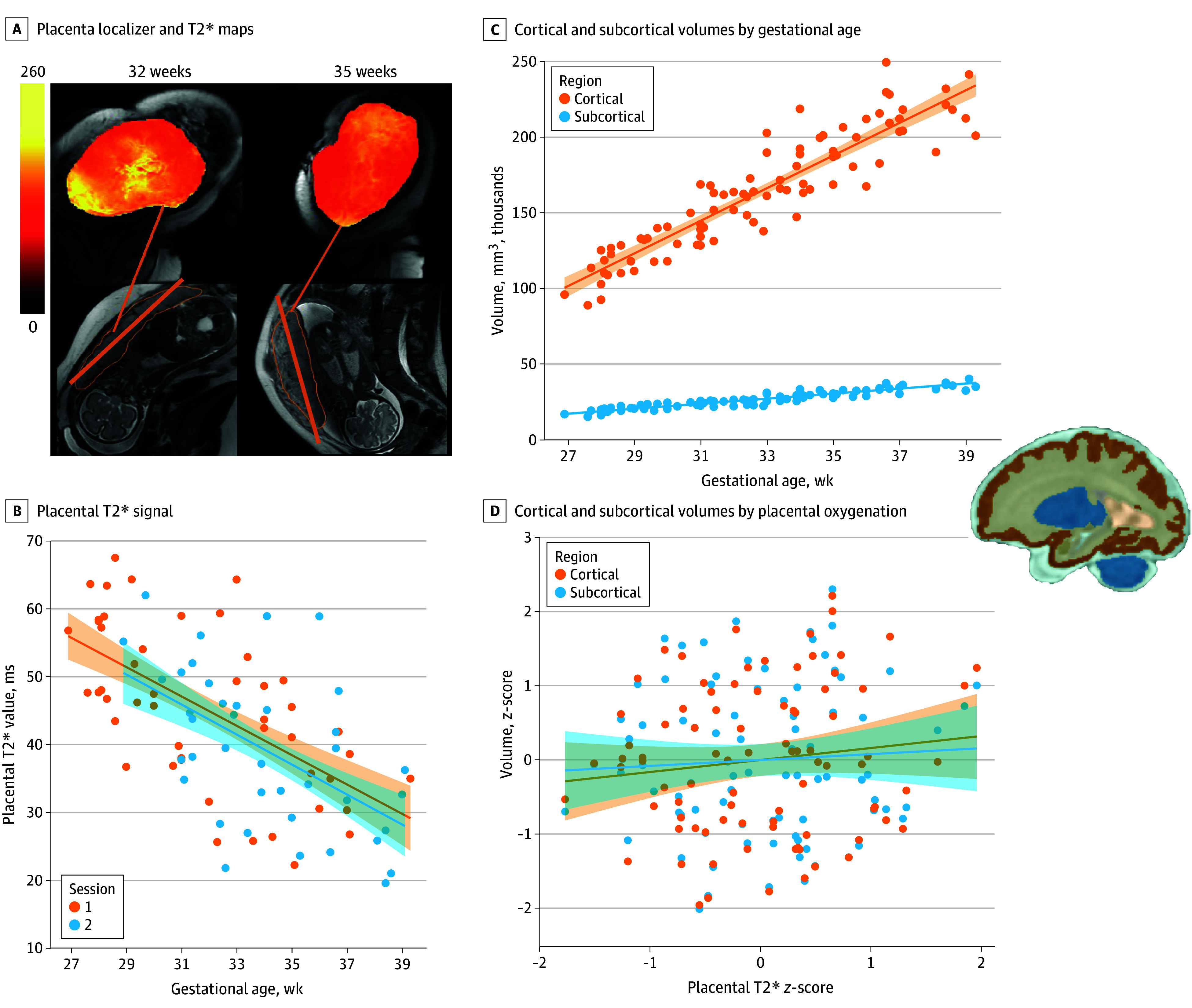
Placental T2* Values and Cortical and Subcortical Volumes A, Placenta localizer and T2* maps in a representative participant at 32 (time point 1, a) and 35 weeks (time point 2, b) of gestation. B, Placental T2* signal (time point 1 [n = 49]; time point 2 [n = 38]) in relation to gestational age in weeks at time point 1 (orange) and 2 (blue). C. Cortical and subcortical volumes in relation to gestational age in weeks. D. Cortical and subcortical volumes by placental oxygenation (T2*). Both volumes and T2* values have been *z*-scored. Shaded regions indicate 95% CIs and are based on the mean.

## Discussion

This cohort study found an association between T2* mapping of placental oxygenation and fetal brain volumes. Importantly, this dissociation between T2* and cortical vs subcortical brain regions suggests that the cortex is susceptible to variability in oxygenation levels, demonstrating the feasibility of using T2* as a measure of placental function and fetal hypoxia and a biomarker for fetal brain development. Limitations included a small sample size and selection bias due to the community-based sample.

## Notes

Supplement 1. **eMethods.** Supplementary Methods
eReferences

Supplement 2.
Data Sharing Statement
