# Supplementary material for: T2* Mapping of Placental Oxygenation to Estimate Fetal Cortical and Subcortical Maturation
Source: JAMA Netw Open. 2024 Feb 27;7(2):e240456. doi: 10.1001/jamanetworkopen.2024.0456 (PMC10900962; doi:10.1001/jamanetworkopen.2024.0456)
Supplement: Supplement 1. — eMethods. Supplementary Methods eReferences [file jamanetwopen-e240456-s001.pdf]

Supplemental Online Content

Nichols ES, Al-Saoud S, de Vrijer B, et al. T2* mapping of placental oxygenation to estimate fetal cortical and subcortical maturation. *JAMA Netw Open.* 2024;7(2):e240456. doi:10.1001/jamanetworkopen.2024.0456

Supplement 1. **eMethods.** Supplementary Methods

Supplement 2. **Data Sharing Statement**

eReferences

This supplemental material has been provided by the authors to give readers additional information about their work.

© 2024 Nichols ES et al. *JAMA Network Open.*

eMethods. Supplementary Methods

MRI protocol: 2D multi-echo stack imaging parameters were acquired using the following sequence: TR=81.1 ms, 16 echoes ranging from 3.15 to 77.31 ms in steps of 4.9. Three single shot fast spin echo images (repetition time>1,000 ms, echo time=80 ms, field of view=38–44 mm, 0.74 x 0.74 [coronal and sagittal] or 0.86 x 0.86 [axial] matrix, slice thickness=5 mm, 19–25 slices) were obtained, with one in each *x*, *y*, and *z* plane. The resulting brain images were corrected for motion and bias field inhomogeneities, and super-resolution reconstruction was performed, resulting in 3D volumes resampled to 1 mm isotropic resolution. Following 3D reconstruction, nonlinear registration of the anatomical image to a 36-week gestational age fetal brain template (Gholipour et al. 2017) was performed using ANTs. This atlas comes with 126 predefined labels. Using the inverse of the native-to-template transformation matrix, the labeled atlas was transformed into native space for each participant. Region of interest (ROI) masks were then generated for cortical and subcortical regions using AFNI's *3dCalc*, and volume information was extracted for each ROI using FSL's *fslstats*.

Statistical analysis: Z-scores for T2* values were calculated to adjust for gestational age as previously reported by Sinding et al. (2018). First, a regression model was fit to estimate T2* values from gestational age in weeks. The predicted T2* value from the model was then subtracted from the actual T2* value, divided by the standard deviation of the predicted values. Z-scores for cortical and subcortical volumes were calculated separately, by subtracting the mean cortical or subcortical volume from the actual value, and dividing by the standard deviation. This was performed in order to be able to accurately compare cortical and subcortical volumes, which as raw values are on very different scales (see Figure 1D).

© 2024 Nichols ES et al. *JAMA Network Open.*

eReferences

Gholipour A, Rollins CK, Velasco-Annis C, Ouaalam A, Akhondi-Asl A, Afacan O, Ortinau CM, Clancy S, Limperopoulos C, Yang E, et al. (2017). A normative spatiotemporal MRI atlas of the fetal brain for automatic segmentation and analysis of early brain growth. *Scientific Reports*, *7*(1), 1–13.

Sinding M, Peters DA, Poulsen SS, Frøkjær JB, Christiansen OB, Petersen A, Uldbjerg N, Sørensen A. Placental baseline conditions modulate the hyperoxic BOLD-MRI response. Placenta. 2018 Jan;61:17-23. doi: 10.1016/j.placenta.2017.11.002.
