# Supplementary material for: T2* Mapping of Placental Oxygenation to Estimate Fetal Cortical and Subcortical Maturation
Source: JAMA Netw Open. 2024 Feb 27;7(2):e240456. doi: 10.1001/jamanetworkopen.2024.0456 (PMC10900962; doi:10.1001/jamanetworkopen.2024.0456)
Supplement: Supplement 2. — Data Sharing Statement [file jamanetwopen-e240456-s002.pdf]

Data Sharing Statement

Nichols. T2* Mapping of Placental Oxygenation to Estimate Fetal Cortical and Subcortical Maturation. *JAMA Netw Open*. Published February 27, 2024.
doi:10.1001/jamanetworkopen.2024.0456

Data
Data available: Yes
Data types: Deidentified participant data
How to access data: The processed data are available upon reasonable request to Emily Nichols (enicho4@uwo.ca) or Emma Duerden (eduerden@uwo.ca).
When available: With publication

Supporting Documents
Document types: None

Additional Information
Who can access the data: Researchers whose proposed use of the data has been approved.
Types of analyses: For any research purpose.
Mechanisms of data availability: With a signed data access agreement.
